# Interfacial Interaction in MeO_x_/MWNTs (Me–Cu, Ni) Nanostructures as Efficient Electrode Materials for High-Performance Supercapacitors

**DOI:** 10.3390/nano14110947

**Published:** 2024-05-28

**Authors:** Galina E. Yalovega, Maria Brzhezinskaya, Victor O. Dmitriev, Valentina A. Shmatko, Igor V. Ershov, Anna A. Ulyankina, Daria V. Chernysheva, Nina V. Smirnova

**Affiliations:** 1 Faculty of Physics, Southern Federal University, 344090 Rostov-on-Don, Russia; vdmitriev@sfedu.ru (V.O.D.); shmatko86@mail.ru (V.A.S.); 2 Helmholtz-Zentrum Berlin für Materialien und Energie, 12489 Berlin, Germany; maria.brzhezinskaya@helmholtz-berlin.de; 3 Department of Physics, Don State Technical University, 344000 Rostov-on-Don, Russia; iershov@donstu.ru; 4 Research Institute “Nanotechnologies and New Materials”, Platov South-Russian State Polytechnic University, 346428 Novocherkassk, Russia; anya-barbashova@yandex.ru (A.A.U.); da.leontyva@mail.ru (D.V.C.); smirnova_nv@mail.ru (N.V.S.)

**Keywords:** MeO_x_/MWNTs nanostructures, interfacial interaction, supercapacitors, XAS, XPS, DFT

## Abstract

Due to their unique physical and chemical properties, complex nanostructures based on carbon nanotubes and transition metal oxides are considered promising electrode materials for the fabrication of high-performance supercapacitors with a fast charge rate, high power density, and long cycle life. The crucial role in determining their efficiency is played by the properties of the interface in such nanostructures, among them, the type of chemical bonds between their components. The complementary theoretical and experimental methods, including dispersion-corrected density functional theory (DFT-D3) within GGA-PBE approximation, scanning electron microscopy (SEM), X-ray diffraction (XRD), Raman, X-ray photoelectron, and X-ray absorption spectroscopies, were applied in the present work for the comprehensive investigation of surface morphology, structure, and electronic properties in CuOx/MWCNTs and NiO_x_/MWCNTs. As a result, the type of interfacial interaction and its correlation with electrochemical characteristics were determined. It was found that the presence of both Ni–O–C and Ni–C bonds can increase the contact between NiO and MWCNTs, and, through this, promote electron transfer between NiO and MWCNTs. For NiO_x_/MWCNTs, better electrochemical characteristics were observed than for CuO_x_/MWCNTs, in which the interfacial interaction is determined only by bonding through Cu–O–C bonds. The electrochemical properties of CuO_x_/MWCNTs and NiO_x_/MWCNTs were studied to demonstrate the effect of interfacial interaction on their efficiency as electrode materials for supercapacitor applications.

## 1. Introduction

Supercapacitors are promising electrochemical energy storage devices due to their high power density, excellent cycle efficiency, fast charge/discharge rate, reliability, and tunable characteristics. Complex nanostructured materials based on both copper and nickel oxides in combination with carbon nanotubes (CNTs), graphene, and graphene oxides make it possible to create supercapacitors with desired characteristics, such as a fast charge rate, high power density, and long cycle life [1,2,3,4,5].

NiO and Ni(OH)_2_ are typical pseudocapacitor electrode materials with a relatively high capacitance but low cycling and electron transport lifetime [6]. CuO is also considered an effective electrode material for supercapacitor applications due to its narrow bandwidth and pseudocapacitor nature [7]. Combining them with carbon nanostructures in the same composite can provide a higher specific surface area and better electronic conductivity due to better charge transfer. Charge transfer has a positive effect on the electrochemical performance of composites based on carbon materials, such as carbon nanotubes (CNTs) and graphene with transition metal oxides. In the case of reduced graphene oxide/carbon nanotubes/NiO composites [8] CNTs prevent RGO/NiO aggregation and improve electron transport, thereby demonstrating significantly enhanced electrochemical performance. In [9], it has been demonstrated that the interaction of nickel with reduced graphene oxide, as well as nickel oxide with the walls of carbon nanotubes in the NiO@srGO/CNT, increases the efficiency of interfacial transfer and improves electrochemical properties. In [10], the good capacitance of CuO/rGO is attributed to reduced graphene oxide nanosheets that provide a better electronic connection between CuO nanoparticles initiating electron transfer. In [11], electron transport promoted by metal doping and support was observed in Co-doped NiO nanosheets on a carbon nanotube framework. 

One of the factors influencing the efficiency of such electrode materials is the characteristics of the interfacial surface and interfacial interaction [12]. Morphology, composition, local atomic and electronic structure, molecular and crystallographic orientation, as well as the type of chemical bonds between components determine the electrical, catalytic, and adsorption properties of the resulting materials [13,14,15,16,17,18]. The influence of the kind of matrix on directing the nano-unit nucleation and subsequent organization of metal oxide crystallites is observed [19]. The reason for this phenomenon is the interaction between the carbon matrix and metal oxide nanoparticles, leading to changes in the local atomic and electronic structure of both components of the resulting composite and, as a consequence, changes in the properties of the whole composite. Thus, the electrochemical and adsorption properties of metal oxides can be tuned by tuning their particle size, crystallinity, and morphology [20,21]. Electrochemical tests have shown that the specific capacitance of the nanocomposite NiO@CNTs can be twice that of the pure NiO because CNTs increase the surface area of NiO and provide a conducting pathway to facilitate a fast electrochemical process [2]. Asymmetric supercapacitors fabricated from Ni(OH)_2_/oxidized CNT nanocomposite exhibited significant capacitance compared to pure Ni(OH)_2_ and oxidized CNT due to the fact that the Ni(OH)_2_ was coated coaxially to the CNT surface, which could greatly improve matrix conductivity [22]. Electrode NiMoO_4_–CNTs–CuO exhibited a high specific areal capacitance which was ascribed to the synergistic effects of binary metal oxide, CNTs’ conductive networks, and their core-shell nanostructure [23]. All these results indicate that CNTs are suitable for improving electron transport in composite electrodes. 

The actual problem in the research of nanocomposites based on sp^2^ nanocarbon materials (CNT, graphene, etc.) and different metals or their oxides/hydroxides is the investigation of the interaction mechanisms of the CNT surface with a metal-containing component, the chemical state of the carbon atoms in the CNT outer wall, and peculiarities of the structure and chemical composition of the CNT-metal oxide interface. Understanding the features of interfacial interaction opens the way to the possibility of forming materials with given structural and morphological features and physico-chemical characteristics. As a rule, the metal-containing component of nanocomposites is a mixture of metal oxides, and, therefore, the most important question is whether the carbon atoms of the CNT wall bind directly to the metal atoms, forming a metal-carbon bond, and/or through an oxygen atom, forming a metal-oxygen-carbon bond [24]. 

Efficient methods to study the interaction mechanisms of composite materials components are core level spectroscopy methods—the NEXAFS (near edge X-ray absorption fine structure) or XANES (X-ray absorption near edge structure) method—which allow for the study of the local atomic and electronic structure around the absorbing atom and X-ray photoelectron spectroscopy, and the XPS method. XAS is a local and site-sensitive technique providing information about both the electronic and atomic structures around the absorbing center. Together, XANES/NEXAFS and XPS can provide information about the geometry of the absorbing atom [25] and structural defects [26], and, being elementally selective, they provide information about the chemical composition, presence of elements and functional groups on the surface [27,28,29,30], the oxidation state of elements [29,31,32], and the nature of chemical bonding between the CNT and MeO_x_ causing the formation of the composite [33]. Analysis of up-to-date publications shows that the theoretical interpretation of XANES spectra of nanocomposites based on transition metal oxides and carbon nanotubes is rarely used due to the complexity of applying this technique to the study of nanocomposites. However, the theoretical analysis of the experimental XANES spectra can be used to investigate the mechanisms of interaction between the carbon and metal-containing components in composite systems [34,35]. 

In the proposed paper, we set a goal to come closer to an understanding of the influence of the interfacial interaction on the electrochemical characteristics of the MeO_x_/MWNTs (Me–Cu, Ni) nanostructures. Complementary experimental methods, including the use of synchrotron radiation, and theoretical methods, including DFT and theoretical analysis XANES spectra, were applied to determine the type of interfacial interaction between MWCNTs walls and MeO_x_ (Me–Cu, Ni) nanoparticles. The combination of experimental and theoretical methods, which allow us to follow the changes of atomic and electronic structure both on the surface and in the depth of the samples, made it possible to show that the mixed type of interfacial interaction (covalent and non-covalent) can promote electron transfer and improve electrophysical characteristics. Methodological approaches to the interpretation of the experimental data of such complex nanostructures as MeO_x_/MWNTs (Me–Cu, Ni) have been developed in the present paper. Here, we applied density functional theory (DFT) to simulate the structural properties of MeO_x_/CNT interfaces. DFT can be used not only to study the structure and properties of various interfaces but also to model their formation (utilizing DFT-based so-called Synthetic Growth Concept) [36,37]. We have shown that the theoretical analysis of XANES spectra can provide important information taking into account the different geometries of the surroundings of the absorbing atom—on the particle surface and at the MeO_x_–CNT interface. Comparison of experimental spectra with a linear combination of theoretical spectra of different models gives much more accurate information and allows us to understand the spectrum-composition-structure relationship. Electrochemical measurements were involved as model measurements to illustrate the correlation of electrochemical properties of heterogeneous systems based on MWCNTs and MeO_x_ nanoparticles with composition and interfacial interaction types. Taken together, the results obtained reflect the influence of interfacial interaction on the applied electrochemical properties of electrode materials, making an additional contribution to the knowledge and understanding of the fundamental triangle “composition-structure-properties”.

## 2. Materials and Methods

The NiO_x_/MWCNTs and CuO_x_/MWCNTs nanostructures were prepared by the method based on electrochemical oxidation and dispersion of metal under pulse alternating current in alkaline solutions. The Ni and Cu electrodes were immersed in a suspension of MWCNTs in a solution of 2 M NaOH or 2 M NaCl, respectively. Highly purified commercial (LG Chem, Seoul, Republic of Korea) multiwalled carbon nanotubes with an average diameter of 5 nm, a length of 100 μm, and a specific surface area of 274 m^2^g^−1^ degassed at 300 °C for 2 h in a helium atmosphere were added to the solution. For NiO_x_/MWCNT preparation, an asymmetric pulse alternating current (AC) with the amplitude ratio of the anode and cathode pulses j_a_:j_c_ = 1:4 (j = 0.13:0.52 A cm^−2^) was used. To prepare the CuO_x_/MWCNTs, the density of a symmetric current was 1.0 A cm^−2^ with an amplitude ratio of the cathode and anode pulses of j_a_:j_c_ = 1:1 (j = 1 A cm^−2^). The content of oxides in both nanostructures was regulated by the duration of synthesis and amounted to 10 wt.%.

Morphological study of the CuO_x_/MWCNTs and NiO_x_/MWCNT nanostructures was carried out using a LEO 1560 (ZEISS, Jena, Germany) scanning electron microscope (SEM) at the acceleration voltage of 5 kV and Nova NanoLab 600 (FEI Company, Eindhoven, The Netherlands) at the acceleration voltage of 10 kV, respectively. 

The C K-edge, Ni, and Cu L_2,3_-edge NEXAFS spectra were recorded in the total electron yield detection mode [38] (TEY) at the Russian-German Laboratory Beamline (RGBL) of BESSY II synchrotron radiation facility (Helmholtz-Zentrum Berlin, Germany) with the experimental station equipped with the Phoibos-150 spherical analyzer (SPECS) [39]. The energy resolution was 0.07 eV near the C K-edge and ~0.5 eV near the Cu L_2,3_-edge and the Ni L_2,3_-edge [40]. X-ray photoelectron spectra in the C 1s range were recorded in the normal photoemission registration mode at hv = 1030 eV with an energy resolution of 0.2 eV. All X-ray photoelectron spectra were fitted by the Gaussian/Lorentzian convolution functions with simultaneous optimization of the background parameters using UNIFIT2015 software. The Ni and Cu K-edge XANES spectra were measured in the fluorescence mode at the hard X-ray beamline KMC-2 of BESSY II. The stored beam current was ~280 mA, the slit width was set to 800 μm, and the size of the focused spot on the sample was ~0.2 × 0.1 mm. Additionally, the spectra of copper and nickel foils were recorded simultaneously with the absorption spectra of the nanostructures. The spectra were processed (background subtraction, normalization) using the Athena software version 0.8.56 [41]. 

Raman analysis of the MWCNTs and MeO_x_/MWNTs nanostructures was performed on Renishaw using a Via Reflex Raman spectrometer (Renishaw plc, Wotton-under-Edge, UK) with 325 nm and 514 nm lasers. 

X-ray diffraction (XRD) measurements were performed using step scan mode with a step width of 0.01 and step time of 2.00 s by ARL X’TRA Powder Diffractometer ((Thermo Electron SA, Ecublens, Switzerland) with a Cu Ka radiation source (λ = 1.5418 Å). 

Electrochemical studies were carried out with potentiostat—galvanostat P-50 Pro (Elins, Chernogolovka, Russia) in a standard three-electrode cell configuration. Pt and Ag/AgCl wires were used as the counter electrode and reference electrode, respectively. The working electrodes were prepared by the procedure described in [42]: 15 mg of a MeO_x_/MWCNTs powder (Me = Cu or Ni) was suspended in a mixture of 2-propanol (Sigma-Aldrich, St. Louis, MI, USA) and 10 wt.% aqueous Nafion^TM^ solution (Sigma-Aldrich). The mixture was stirred with a magnetic stirrer and then the formed ink was dropped on a glassy carbon plate electrode (Sigma-Aldrich) and was air-dried. The amount of dried ink in the working electrode was 1 mg. The measurements were carried out by cyclic voltammetry and the galvanostatic charge-discharge technique in a 1 M NaOH aqueous electrolyte solution for the NiO_x_/MWCNTs composite and in 1 M Na_2_SO_4_ for the CuO_x_/MWCNTs.

Self-consistent first-principles calculations were performed using the Density functional theory within the plane-wave pseudopotential method [43]. To consider the electron exchange-correlation interaction, the generalized gradient approximation (PBE functional) was used. The ultrasoft pseudopotentials were used to represent electron-ion interaction. Non-covalent long-range interactions between the nanotube and the oxide surfaces were taken into account using the semi-empirical dispersion-corrected DFT-D3 scheme. The DFT-D3 method, proposed by Grimme [44] to model dispersion interactions, was chosen due to its high accuracy, relatively good computational efficiency, and less empiricism compared to overparameterized empirical and non-local density functionals and dispersion-corrected atom-centered potentials. The interaction of a nanotube with NiO, Ni(OH)_2_, CuO, and Cu_2_O nanoclusters was studied from the first principles using original models within the supercell approach. In accordance with the XRD data, we constructed supercells based on a periodic metal oxide slab five atomic layers thick interfaced with a fragment of a 5 nm armchair nanotube (37, 37) periodic in one dimension. The following crystallographic surfaces were considered: NiO (200), CuO (110), Ni(OH)_2_ (001), and Cu_2_O (110). The nanotube fragment was passivated by hydrogen and was fitted with the oxide unit cell in order to obtain a lattice mismatch lower than 4%. The nanotube fragment was considered as a rigid body with fixed atomic positions, while the positions of the atoms in the top three interface layers were fully optimized with the force threshold of 2 × 10^−3^ Ry/Å. The initial distances between the nanotube fragment and oxide surfaces were calculated using classical molecular dynamics (MD) with the COMPASS II forcefield [45]. The microcanonical (NVE) ensemble was used in the MD run with a time step of 1 fs, the initial temperature was set to 298 K, and the overall duration of the run was 5 ps. The interfaces were separated from their periodic images in the supercell by a vacuum layer of at least 12 Å. The following crystal structures of bulk oxides [46] were used: cubic NiO with lattice constant a = 4.168 Å; hexagonal Ni(OH)_2_ with lattice constants a= 3.125 Å, c = 4.473 Å, g = 120.0; monoclinic CuO with lattice constants a = 4.563 Å, b = 3.41 Å, and c = 5.108 Å, b = 99.48; cubic Cu_2_O with lattice constant a = 4.247 Å.

The Kohn-Sham orbitals have been expanded in plane waves up to a kinetic energy cutoff of 35 Ry. The kinetic energy cutoff for charge density and potential was 280 Ry. The SCF convergence threshold was 10^−6^ Ry. The k-point mesh included only the gamma point of the Brillouin zone.

The interaction in the metal oxide-nanotube interface was characterized by the adsorption energy using the following relation:

$$E_{\mathrm{ads}}=\left(E_{\mathrm{MO}/\mathrm{CNT}}-E_{\mathrm{MO}}-E_{\mathrm{CNT}}\right)/n,$$

where E_MO/CNT_ is the total energy of the metal oxide-nanotube interface, E_MO_ and E_CNT_ are the total energies of metal oxide slab and nanotube fragment, respectively, n is the number of atoms in the eight nearest to the surface zigzag chains of the nanotube fragment.

Ab initio calculations of Ni and Cu K-edge XANES spectra were performed using the full potential finite difference method implemented in FDMNES [47]. Theoretical spectra were convolved using the Lorentzian shape function. The size of the calculated cluster was 7 Å around the absorber and included 132–150 atoms, depending on the structural model. The contributions of different sites of absorber Me atoms in the system to the spectra of the composites were estimated using a linear combination fitting (LCF). Spectral normalization and LCF were all performed using the Athena software [41]. LCF was performed between −20 and 30 eV from the main peak. 

## 3. Results

### 3.1. SEM and XRD Characterization

**Figure 1:**
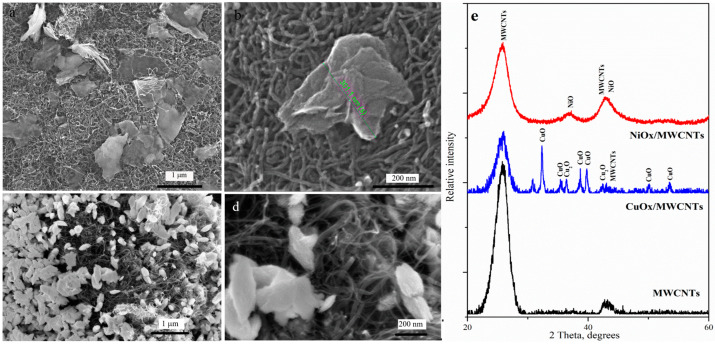
SEM images of nanostructures of (**a**,**b**) NiO_x_/MWCNTs and (**c**,**d**) CuO_x_/MWCNTs. (**e**) XRD MWNTs and MeO_x_/MWNTs nanostructures.

The morphology of the metal oxide component of nanostructure electrode materials significantly affects their electrochemical performance [48,49,50]. SEM images indicated that, during the formation of the NiO_x_/MWCNTs nanostructure, the quite large NiO_x_ nanosheets (average size is about 500 nm) are randomly dispersed among nanotubes (Figure 1a,b). As was shown in [51], nanosheets are composed of agglomerated nanoparticles, which is quite a common phenomenon in the various self-assembled NiO nanostructures. The CuO_x_/MWCNTs nanostructures are characterized by a uniform distribution of numerous flake CuO_x_ nanoparticles, with sizes in the range of 200–500 nm, across the surface of the nanotubes. Figure 1e shows the measured XRD patterns of nanostructures MeO_x_/MWNTs and pure MWNTs. For the NiOx/MWCNTs, the peaks correspond to the planes of cubic phase NiO. For the CuO_x_/MWCNTs, the peaks correspond to the planes of monoclinic CuO and cubic Cu_2_O, which are in good agreement with previous works [52,53].

### 3.2. Raman Analysis

**Figure 2:**
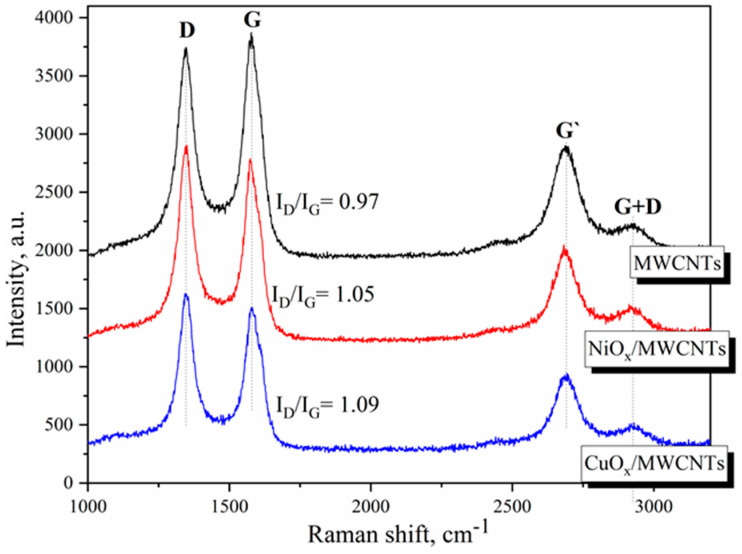
Raman spectra for MWNTs and MeO_x_/MWNTs nanostructures in the range of 1000–3200 cm^−1^.

The structural changes of MWCNTs that occurred during the formation of the nanostructures were determined by changes in the spectral bands of Raman spectroscopy. It can be seen in Figure 2 that all spectra showed a similar D-band (~1345 cm^−1^) and G-band (~1580 cm^−1^). The peak at 1345 cm^−1^ is assigned to the defects or to the sp^3^ hybridized carbons, while the peaks at 1580 cm^−1^ are attributed to the graphite band, which is common to all sp^2^ systems. In addition, all spectra exhibit the G′-band (~2695 cm^−1^), which is the overtone of the D-band [54] and G+D band (2930 cm^−1^).

The I_D_/I_G_ ratio is an indication of the structural defects in graphitic carbons caused by the chemical modification of carbon materials [55]. As can be seen, for nanostructures, redistribution of the intensities of the D and G bands is observed in comparison with the spectra of pure nanotubes. The intensity ratios I_D_/I_G_ obtained after the approximation of the spectra are 0.97, 1.05, and 1.09 for MWCNTs, NiO_x_/MWCNTs, and CuO_x_/MWCNTs, respectively. The increase in the I_D_/I_G_ ratio for nanocomposites may be due to the occurrence of defects induced by oxygen or other atoms on the surface of the nanotubes and a change in C–C hybridization from sp^2^ to sp^3^ [56].

### 3.3. XPS Characterization

**Figure 3:**
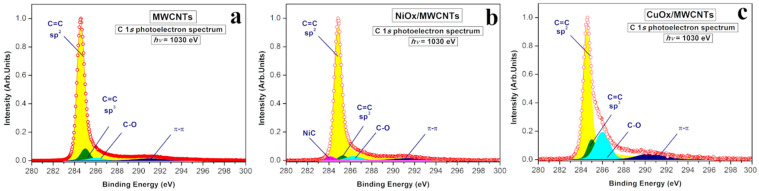
C 1s X-ray photoelectron spectra of the MWCNT (**a**), NiO_x_/MWCNT (**b**), and CuO_x_/MWCNT (**c**) nanostructures measured at hv = 1030 eV.

X-ray photoelectron spectra (XPS) of the initial MWCNTs and MeO_x_/MWCNTs (Ni, Cu) nanostructures were recorded in the vicinity of the narrow C 1s peak at hv = 1030 eV (Figure 3a–c). The C 1s spectrum of the initial MWCNTs sample (Figure 3a) has a complex structure that can be well described by deconvolution with four components. The main component is located at a binding energy of BE = 284.8 eV, which is typical for the C–C graphene-like carbon atoms with the sp^2^-hybridization of the valence electron states. The presence of a π–π* satellite (BE~291.3 eV) also confirms the predominating sp^2^ character of the graphene layers in the initial MWCNTs. The presence of chemisorbed oxygen, in the form of hydroxyl, carbonyl, or carboxyl groups (C–O_x_), leads to the appearance in the C 1s photoelectron spectrum of the additional shoulder at BE = 286.3 eV [57]. However, the large amount of C=C bonding corresponded to sp^2^-hybridization (Table 1), and the relatively small amount of C–C bonding corresponded to sp^3^-hybridization, indicating the high purity of the initial MWCNTs, which is consistent with the NEXAFS spectroscopy data presented below.

It is important to note that, during the formation of a NiO_x_/MWCNT nanostructure, the appearance of a feature at BE = 284.1 eV corresponding to Ni–C bonding is observed [58], while the intensity of the sp^2^—component remains the same as in the initial MWCNTs (Figure 3b). 

The formation of CuO_x_/MWCNTs leads to an increase in the asymmetry coefficient α = 0.16 (in comparison with MWCNTs and NiO_x_/MWCNTs α = 0.1) as well as a decrease in the intensity of the sp^2^-component. At the same time, there is a significant increase in the amount of C–C bonding, corresponding to sp^3^-hybridization, and C–Ox bonding in comparison with MWCNTs and NiO_x_/MWCNTs (Figure 3c). It should be noted that XPS is a surface-sensitive method and the information obtained at hv = 1030 eV is limited to a probing depth of ~1.5 nm. 

### 3.4. NEXAFS Characterization

The type of interaction between MWCNTs and MeO_x_ nanostructures is the most important factor because it can affect conductivity, specific capacity, and rate capability, as was shown for the Ni(OH)_2_/MWCNT hybrid [32].

**Figure 4:**
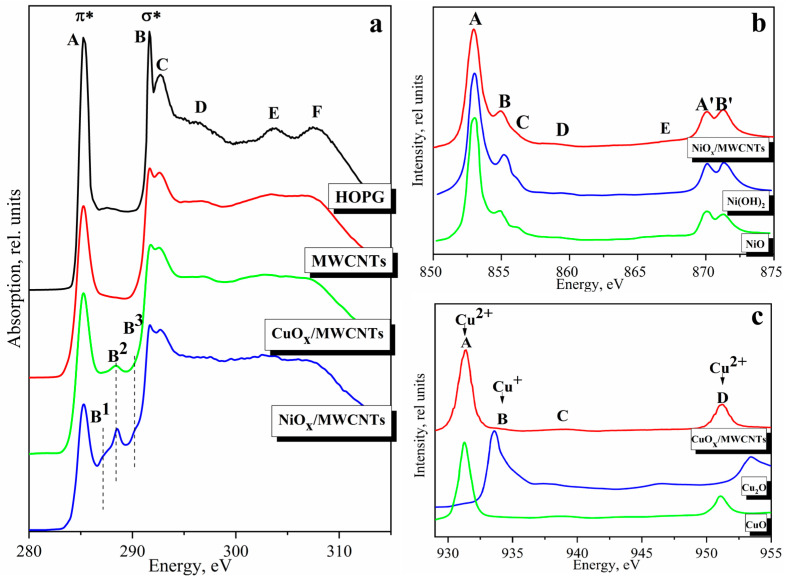
The C 1s NEXAFS spectra of HOPG, MWCNTs, NiO_x_/MWCNTs, CuO_x_/MWCNT (**a**), Ni 2p absorption spectra of NiO_x_/MWCNTs, Ni(OH)_2_ and NiO (all reference spectra from [60]) (**b**), Cu 2p absorption spectra of CuO_x_/MWCNTs, CuO, and Cu_2_O (all reference spectra from [61]) (**c**).

It follows from the comparison of the C 1s NEXAFS spectra of the initial MWCNTs and NiO_x_/MWCNTs, CuO_x_/MWCNT that all spectra reproduce all the main features A–D of the HOPG spectrum (Figure 4a) [59]. The σ resonance peaks (C 1s→σ* transitions) are associated with intramolecular bonds. The double and sharp B–C peaks suggest a well-formed local bonding configuration and well-formed nanotube structure. The energy position of peak A, as well as the shape and energy position of peaks B–C, are the same in the spectra of nanostructures and initial nanotubes, which confirms the structural stability of nanotubes during the synthesis of nanostructures. Some broadening of D–F peaks is observed for the nanostructures compared to HOPG and MWCNT which indicates the appearance of amorphous carbon or a higher extent of defectiveness [34]. The reduction in A-band intensity in the nanostructures demonstrates a reduction in the free π-symmetry states of the conduction band, in contrast to the initial MWCNTs. As we have shown in the case of hydrogenated CNTs, the decrease in resonance intensity is related to an increase in the number of atoms attached to the tube surface [34]. The features in the energy region between 285 eV and 291 eV indicate oxidation or other modifications of the nanotubes. These peculiarities appear due to the transition of C 1s electrons to the free 2p states and correspond to the formation of sp^3^ hybridized electronic states of carbon atoms associated with the adsorbed molecules on the side wall of the nanotubes. The absence of any features in this energy range for the initial nanotubes indicates their high purity (Figure 4a), namely, the absence of functional groups on the surface layers, which is consistent with the XPS data. 

The C 1s NEXAFS spectrum of the NiO_x_/MWCNTs indicates the appearance of the new spectral features B^1^, at ~287.4, B^2^, at ~288.6, and B^3^, at ~290.4 eV, respectively. The peak B^1^, at ~287.4 eV, is associated with the transitions of the C 1s core electrons to π unoccupied orbital of the C–O and C–O–C functional groups [24]. The peak at ~288.6 eV was assigned to π*(CO) resonance and related to the carboxyl functional groups [62]. The peak at ~290.4 eV is due to π-resonance in carbonate [CO_3_]_2_ [32,63]. The increases in peak intensity at ~288.6 eV (carboxyl π*) and 290.4 eV (carbonate π*) were attributed to the formation of Me–O–C bonding via the carboxyl group on the CNT surface in the NiFe–LDH/CNT complex [64] and in the Ni(OH)_2_/MWNT hybrid [32]. 

The CuO_x_/MWCNT has a peak at 288.6 eV (B_2_) which corresponds to carbon-oxygen bonds [65] and is caused by the interaction of nanoparticles with the carbon matrix by covalent sharing of external oxygen electrons of oxide component and matrix carbon [66]. The presence of chemically bound oxygen in nanostructures is also confirmed by the XPS data. Interaction effects between NiO_x_ nanosheets and CuO_x_ nanoparticles with MWCNTs can facilitate charge transfer [64]. A similar phenomenon was observed for the Cu/MWCNTs [67] and was determined by the formation of a Cu–O–C bond in the CuO/(graphene oxide) composite [68], and a Sn–O–C bond in the SnO/MWCNTs [69]. It should be noted that NEXAFS spectroscopy is also a surface-sensitive method, the depth of the signal obtained for C K-spectrum is greater than in XPS but limited to ~15 nm.

The formation of bonds between the carbon atoms in the MWCNTs and the MeO_x_ particles present in the nanostructures, which could be formed as a result of electrochemical oxidation and dispersion of metal, can be explored by analyzing the Ni and Cu 2p absorption spectra. Figure 4b,c shows the comparison of the Ni and Cu 2p absorption spectra of the investigated NiO_x_/MWCNTs and CuO_x_/MWCNTs with reference samples. All spectra consist of two components due to the spin-orbital splitting of the 2p level of the nickel atom into Ni 2p_3/2_ and Ni 2p_1/2_ sublevels [58], and of the copper atom into Cu 2p_3/2_ and Cu 2p_1/2_ sublevels [24]. The 2p_3/2_ spectra for 3d atoms, including Ni and Cu metals, have a more pronounced structure and are currently considered the most direct experimental source of information concerning the distribution of free 3d-states of 3d metal atoms. The structure of the spectra is associated with the dipole transitions of 2p_3/2_ electrons to free electronic states making contributions to the 3d states. 

As can be seen from Figure 4b, the Ni 2p absorption spectrum of NiO_x_/MWCNTs exhibits all the main details typical for the absorption spectra of Ni(OH)_2_ and NiO. The splitting between peaks A and B for the NiO_x_/MWCNTs sample is 2.02 eV, which is higher than that of NiO (~1.8 eV), and exactly coincides with Ni(OH)_2_ (~2.0 eV). Peak A’ (Ni 2p_1/2_ component) in the spectrum of NiO_x_/MWCNTs is separated from peak A by 17.10 eV, which is less than in the NiO spectrum (17.13 eV) and coincides with the similar value in the Ni(OH)_2_ spectrum (17.10 eV). The difference in the positions of the peaks A’ and B’ in the spectrum of the sample NiO_x_/MWCNTs (1.2 eV) is closer to the similar value for Ni(OH)_2_ (1.2 eV), but not for NiO (1.17 eV). In other words, the spin-orbit splitting value for the nanocomposite spectra is slightly lower than that of NiO oxide and corresponds to the value for Ni(OH)_2_. At the same time, it should be noted that the intensity ratio of peaks A and B in the spectrum of the nanocomposite sample differs from that in Ni(OH)_2_ and is closer to that of NiO. The Ni 2p spectrum of NiO corresponds to 2p^6^3d^8^→2p^5^3d^9^ transitions, and Ni cations are assigned to Ni^2+^ [70]. It should be noted that the structure of the absorption spectra, namely, the number of features and their energy position, are very similar to the spectra of Ni(OH)_2_ and NiO. Therefore, it is difficult to state unequivocally the predominance of one or another phase in the spectrum of the nanostructures. However, this does not contradict the fact that the nanostructure XRD pattern demonstrates that Ni oxides are mainly composed of NiO. It should be taken into account that XRD is a bulk-sensitive method, while NEXAFS and XPS characterize the composition on the sample surface. The Ni 2p absorption spectrum of NiO_x_/MWCNTs gives us information about the local structure at a depth of ~6 nm.

As can be seen from Figure 4c, the Cu 2p absorption spectrum of CuO_x_/MWCNTs is dominated by intense absorption bands of CuO. However, the appearance of peak B in the spectrum of the nanostructure at an energy of ~933.7 eV refers to Cu_2_O. Thus, the comparative analysis of the Cu 2p NEXAFS spectra shows the presence of CuO and Cu_2_O oxides on the CuO_x_/MWCNTs surface. Moreover, the contribution of CuO is much larger than that of Cu_2_O.

### 3.5. Theoretical Simulation of XANES Spectra

We have calculated the formation energy of the interfaces as the difference between the total energy of the corresponding interface and the sum of the total energies of the relaxed oxide slab and the nanotube fragment. All the obtained values were negative, which indicates that the formation of the interfaces is energetically favorable. Table 2 provides information on adsorption energies and minimal distances between the nanotube and oxide surface in the NiO(200)–CNT, Ni(OH)_2_(001)–CNT, CuO(110)–CNT, and Cu_2_O(110)–CNT interfaces.

The calculated adsorption energies are in agreement with previous theoretical calculations [71,72] and indicate predominantly non-bonding dispersion interaction between carbon nanotube and metal oxides. Previously, it was shown that in metal oxide-graphene composites graphene agglomeration weakens due to van der Waals interaction with the metal oxide surface, resulting in excellent electrochemical performance [73]. Physisorption of metal oxide clusters on the nanotubes, on the one hand, allows for the retaining of excellent electronic and electrochemical properties of nanotubes, and, on the other hand, the formation of such an interface can lower Schottky barrier height and reduce contact resistance.

**Figure 5:**
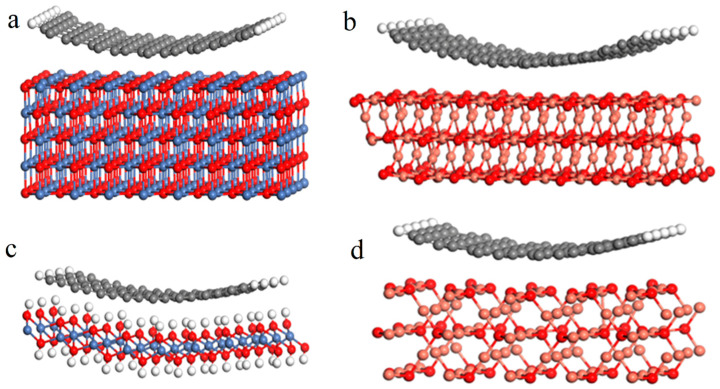
Structural models for calculating theoretical XANES K-edge spectra: orientation to the tube wall plane NiO (200) (**a**), Ni(OH)_2_ (001) (**b**), CuO (110) (**c**), and Cu_2_O(110) (**d**). Blue ball—nickel, pink ball—copper, red ball—oxygen, white ball—hydrogen.

**Figure 6:**
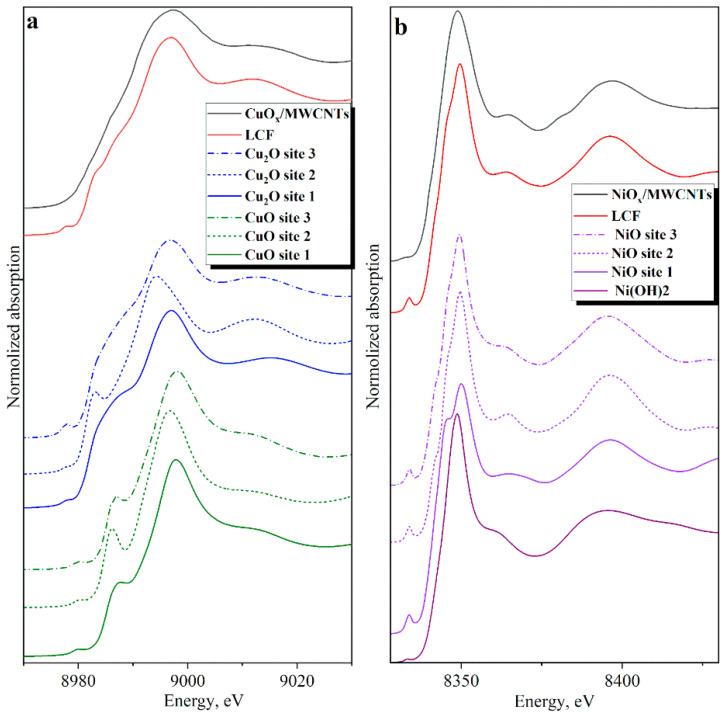
Comparison of experimental and theoretical K-edge XANES spectra: CuO_x_/MWCNTs (**a**) and NiO_x_/MWCNTs (**b**).

A comparative analysis of the interaction of different crystallographic planes with the nanotube graphene layer has been carried out using NiO-CNT and CuO-CNT interfaces as examples. Structural models of NiO(200)–CNT, Ni(OH)_2_(001)–CNT, CuO (110)–CNT, and Cu_2_O(110)–CNT interfaces have been created (Figure 5), and a theoretical simulation of Ni and Cu K-edge XANES spectra for them has been performed (Figure 6). The results are compared with the experimental XANES spectra of the NiO_x_/MWCNTs and CuO_x_/MWCNTs nanostructures. The structural models used for the simulations reflect some important properties of the real structures of CuO_x_/MWCNT and NiO_x_/MWCNT composites based on the experimental data presented above. A comparative analysis of NEXAFS spectra allows us to make an assumption about the local environment of absorbing atoms (See Section 3.4). Thus, a comparison of the Cu 2p NEXAFS spectrum of the CuO_x_/MWCNTs composite with references shows the presence of the copper oxides CuO and Cu_2_O in the composite. The Ni 2p NEXAFS spectrum of NiO_x_/MWCNTs reflects the local environment of nickel, corresponding to NiO and Ni(OH)_2_. The possible formation of bonds between carbon atoms in MWCNTs and MeO_x_ particles is confirmed by analyzing the C 1s NEXAFS spectra of MWCNTs, NiO_x_/MWCNTs, and CuO_x_/MWCNTs. The models do not take into account the difference in the sizes of crystals of oxides and nanotubes (the average crystal size is up to 500 nm, the average nanotube diameter is 5 nm) and the defects of the carbon nanotube. Additionally, it should be noted that other interface configurations may exist. These models represent some modes of interaction between particles and tubes. However, the good agreement between theoretical simulation and the experimental Ni and Cu K-edge XANES spectra (see below) suggests that the main interaction can be described by the interface models we consider in this study.

The formation of spectral features of XANES spectra and the energy position of the absorption edge, reflecting the oxidation state of the absorbing atom, depends on a complex combination of many factors. When analyzing the X-ray absorption spectra, it is important to take into account the different geometry of the absorbing atom’s environment—on the particle surface and at the MeO_x_–CNT interface— and the fact that the local environment of the metal atom differs from that in the volume. Thus, the comparison of experimental XANES spectra of nanostructures with bulk standards of metals and metal oxides does not provide an objective understanding of the change of local structure in composites, whereas the comparison of experimental spectra with a linear combination of theoretical spectra of different models gives much more accurate information and provides an understanding of the spectrum-composition-structure relationship [74].

As input data for the calculations of theoretical absorption spectra, interface models were taken with several metal atoms in different non-equivalent positions—at the MeO_x_–CNT interface (site 1), inside and on the surface of the metal-oxide slab (site 2), and (site 3), respectively—as the absorber. The structural model CuO(110)–CNT used in the calculations of this interface includes carbon atoms in the nearest surroundings of the absorbing copper (site 1) at a distance of 3.11 Å. The structural model Cu_2_O (110)–CNT used in the calculations of this interface includes carbon atoms in the nearest surroundings of the absorbing copper (site 1) at a distance of 3.28 Å. The structural model NiO (200)–CNT used in the calculations of this interface includes carbon atoms in the nearest surroundings of the absorbing nickel (site 1) at a distance of 2.6 Å. The structural model Ni(OH)_2_ (001)–CNT used in the calculations of this interface includes carbon atoms in the nearest surroundings of the absorbing nickel at a distance of 4.27 Å. Figure 6 shows the original calculated XANES spectra and LCF to the bulk for each supported cluster in this study. All spectra are vertically shifted for clarity. As expected, the shape of the spectra depends on the chosen position of the absorbing atom and is very different for internal (site 2) and surface (site 1 and site 3) atoms. As can be seen, the LCF at different sites of absorption indicates that the Me atom fits the experiment better. The obtained R-factor, which is a measure of the mean square sum of the misfit at each data point, is 0.001612 and 0.005270 for CuO_x_/MWCNTs and NiO_x_/MWCNTs, respectively. The LCF for the NiO_x_/MWCNTs gives the following composition: 44.4% site 1 of Ni atom in NiO(200)–CNT model, 43.0% site 2 of Ni atom in NiO(200)–CNT model, 12.6% Ni(OH)_2_, suggesting that the dominant Ni state in the composite is NiO, which is in agreement with the diffraction data. This result is an indirect confirmation of the interaction of NiO particles with the tube through the non-covalent interaction of Ni–C and the nearest surroundings of the absorbing nickel, including carbon atoms (site 1), at a distance of 2.67 Å. However, it should be noted that this crystallographic plane on the surface has both nickel and oxygen atoms and Ni–O–C bonds are probably also formed. The simultaneous presence of Ni–C and Ni–O–C for such systems (NDs@rGO) was shown in the work [75]. As we can see from Table 2, the NiO(200)–CNT interface demonstrates the highest adsorption energy and the lowest interfacial distance, which may indicate bond formation. As we can see from the differential charge density for the NiO(200)–CNT interface (Figure S1 in Supplementary Information), there are small electron depletion regions on the top Ni atoms. The averaged electrostatic potential in the NiO(200)/CNT interface (Figure S2 in Supplementary Information) demonstrates a moderate (about 2 eV) reduction in the tunneling barrier between the nanotube and the top NiO layer, which could enhance electron transmission probability [76,77]. However, the contribution of Ni(OH)_2_ is consistent with Ni L-edge XANES data. We assume that a portion of the NiOx particles in composites is coated with nickel hydroxide. 

The LCF for the CuO_x_/MWCNTs gives the following composition: 35.8% site 2 of Cu atom in Cu_2_O(110)–CNT model, 41.0% site 3 of Cu atom in Cu_2_O/CNT model, 12.3% site 1 of Cu atom in CuO/CNT model, and 10.9% site 3 of Cu atom in the CuO(110)–CNT model. These results show that significant contributions to the spectrum are made by surface atoms. Predominantly, at the CuO_x_-nanotube interface, the particles are in the CuO state. In these models, the smaller CuO–CNT interfaces correspond to the carbon-oxygen (nearest flat copper surroundings). The good agreement between the theoretical spectra of these models and the experimental one confirms that the interaction of CuO_x_ particles with the tube most likely occurs through the formation of Cu–O–C bonds; no Cu–C bond is formed.

### 3.6. Electrochemical Characterization

To demonstrate the influence of differences in the structure and interfacial interaction of nanostructures as well as to demonstrate the possible applications of MeO_x_/MWCNTs, we examined their electrochemical properties as electrode materials for supercapacitors.

**Figure 7:**
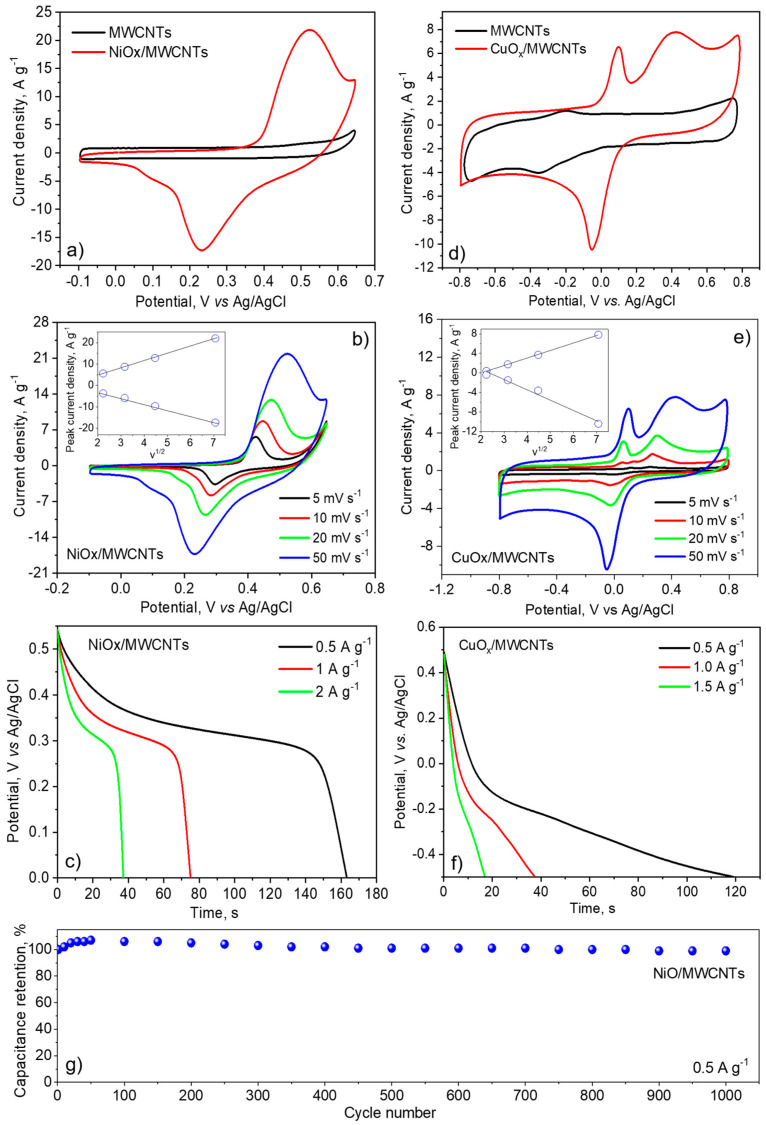
Cyclic voltammetry at potential sweep rate 50 mV s^−1^ of (**a**) NiOx/MWNTs and MWNTs electrodes in 1 M NaOH electrolyte, (**d**) CuOx/MWCNT, and MWCNT electrodes 1 M Na_2_SO_4_. CV curves of NiOx/MWNTs (**b**) and CuOx/MWCNT (**e**) at different scan rates (insert: dependence of the anodic and cathodic peaks current density on the square root of the potential sweep rate). Galvanostatic discharge curves at different current densities of (**c**) NiOx/MWNTs and (**f**) CuOx/MWCNTs materials. Results of long cycling of NiOx/MWNTs in charge/discharge mode at a current density of 0.5 A g^−1^ (**g**).

CV curves at a scan rate of 50 mV s^−1^ of the MWCNTs and NiO_x_/MWCNTs electrodes are shown in Figure 7a. It can be seen that the CV curve shape of the MWCNTs electrode is close to rectangular, which is typical for an electric double-layer supercapacitor (Figure 7a). In contrast, the CV curves of the oxide-containing nanostructures clearly show a quasi-reversible electron transfer process, which indicates that the capacitance of nanostructures is mainly based on the redox mechanism characteristic of pseudocapacitive materials.

With the increase in the scan rate, the CV curves’ shape practically does not change; however, anodic and cathodic peaks shift toward positive potential and negative potential, respectively (Figure 7b). The increase in the current density with the increase in scan rate is linear (insert in Figure 7b) and indicates a good rate capability of the NiO_x_/MWCNTs material as well as the fact that the process of higher nickel oxide formation is controlled by diffusion.

The charge/discharge performance of the NiO_x_/MWCNTs material was investigated by the chronopotentiometry from 0 to 0.55 V and the corresponding results are shown in Figure 7c. The Galvanostatic discharge curve consists of two sections, a sudden potential drop followed by a slow potential decay (Figure 7c). This plateau represents the reversible redox Faradic reactions on the surface of the electrode material:

NiO + OH^−^ ↔ NiOOH + e^−^

The specific capacitances (C_s_) of both nanostructures were calculated by the galvanostatic charge/discharge curves (Figure 7c,f), according to the following equation [42]:

$$C_{s}=\frac{I\times t}{m\times V}$$

where I (A) is the discharge current, t (s) is the time of discharge, V is potential window, and m (g) is the mass of active materials in the working electrode.

The specific capacitance of the NiO_x_/MWCNTs is 154, 149, 148 F g^−1^, and 118 F g^−1^ at appropriate current densities of 0.5, 1, 2 A g^−1^, and 5 A g^−1^.

The CV results of the CuO_x_/MWCNT nanostructures (Figure 7d) show that a Faradic charge transfer processes occurred for the CuO_x_, indicating a pseudocapacitance effect. The CV curves exhibited two distinct anodic peaks near to 0.1 V and 0.3 V, and one distinct cathodic peak at 0 V. The anodic peaks can be attributed to the oxidation of Cu_2_O/CuOH to CuO/Cu(OH)_2_. The cathodic peak is related to the reduction of CuO/Cu(OH)_2_ to Cu_2_O/CuOH [78]. The increase in the current response with the scan rate suggests that the kinetics of interfacial faradic redox reactions and the rates of electronic and ionic transport are significantly fast. Figure 7e (inset) shows an almost linear relationship of the anodic and cathodic peaks current with square root of scan rate confirming the diffusion-controlled electrode reaction that is similar to NiO_x_/MWCNTs’ behavior. The values of specific capacities calculated for CuO_x_/MWCNT are 60.0, 37.0, and 25.5 F/g^−1^, at discharge current densities of 0.5, 1.0, and 1.5 A/g^−1^, respectively. The values are consistent with those given earlier in the literature for CuO_x_-based nanocomposites [79].

Given the better electrochemical performance of NiO_x_/MWCNTs, the long cyclability of this material was tested at a current density of 0.5 A g^−1^. As indicated in Figure 7g, after 1000 charge/discharge cycles 99% of its initial specific capacity remained which indicates excellent stability of this nanomaterial.

## 4. Discussion

Four main types of interfacial interactions between heterogeneous systems based on graphitized carbon and oxide nanoparticles can be distinguished [80]. The interaction of the oxide nanoparticle with the defect-free graphene surface is defined by direct physical contact, when nanoparticles or metal oxide layers are formed directly on graphitized carbon. The second and third types are realized in the presence of covalent functionalization of the surface of nanostructured carbon. These types are interfacial interactions in the presence of structural defects (second type) or chemically bonded functional groups (third type) in the surface layers of CNTs, which causes a stronger interaction between the graphene plane and deposited oxide nanoparticles. Active centers are various oxygen-containing functional groups: hydroxyl, carboxyl, and carbonyl groups, as well as structural defects of graphene planes—non-hexagonal carbon rings, edge atoms, vacancies, and larger vacancy clusters. The last type of interfacial interaction is when the surface of a CNT or graphene interacts with metal oxide nanoparticles with the help of bonding chemical agents. In this case, the interaction directly between the surface of the graphitized carbon and the organic agent is non-covalent. During the interaction, the CNT wall structure remains practically unchanged. Slight changes in the atomic and electronic structure of sp^2^-hybridized carbon can be observed only in the area of direct contact with a metal or oxide particle. Nanostructures with non-covalent type of interaction can exhibit higher electrophysical characteristics compared to nanostructures of similar composition but with covalent type of interaction at the interfaces.

The Ni 2p NEXAFS and Ni 1s XANES spectra suggest that the nanosheets in the NiO_x_/MWNTs contain NiO and Ni(OH)_2_. Considering the different depths of the extracted information, it can be assumed that NiO is partially coated with Ni(OH)_2_. Therefore, XRD and XANES (bulk-sensitive methods) indicate the presence of NiO, while NEXAFS (surface-sensitive method) reflects a greater contribution of Ni(OH)_2_. During the formation of the NiO_x_/MWNTs, the structure of the surface graphene layers of nanotubes is retained to a greater extent (the value of sp^2^ in C 1s XPS of MWCNTs and NiO_x_/MWCNTs is the same), but, as we move deeper into the sample, some disordering is observed, the I_D_/I_G_ obtained in the Raman spectra increases from 0.97 to 1.05 for MWCNTs and NiO_x_/MWCNTs, respectively. This may be related to a large number of sp^3^ hybridized electronic states of carbon atoms associated with adsorbed molecules on nanotubes lying deeper, which is also confirmed by C 1s NEXAFS data. The appearance of carboxyl groups on the surface of nanotubes involved in the electrochemical synthesis of the complex nanostructures may be related to the oxidation of their surface in a sufficiently concentrated alkaline electrolyte solution. According to the XPS data, Ni–C bonds form on the surface of the nanostructures, which may indicate a non-covalent interaction between the nanoparticles and the tube wall, similar to [58]. This assumption is also confirmed by theoretical modeling: the averaged electrostatic potential in the NiO(200)/CNT interface demonstrates a moderate (about 2 eV) reduction in the tunneling barrier between the nanotube and the top NiO layer, while simulation of the XANES spectra shows the existence of C in the local environment of Ni. The rather large size (~500 nm) of the nanosheets and their random distribution among the nanotubes seems also suggestive of the absence of strong covalent bonds. Therefore, it would be possible to assume the implementation of the fourth type of interfacial interactions in its pure form. However, the presence of a large number of carboxyl groups on the surface of the tubes, as well as the fact that the crystallographic plane (200) on the surface has both nickel and oxygen atoms, means that the simultaneous presence of Ni–C, Ni–O–C for such systems seems to be possible. Thus, we assume that the fourth type of interfacial interactions, adding the third type, are realized in NiOx/MWCNTs. As was shown in [81], the electrochemical properties of the electrodes improved as the concentration of the carboxylic groups increased. The difference in the surface yield performance of NiO (200) and carbon nanotube leads to electron transfer at the interface. As shown by SEM measurements for NiO_x_/MWCNTs, the carbon nanotubes in it intertwine with each other and with NiO_x_ particles, and NiO_x_/MWCNT heterogeneity can act as a conducting path, accelerating electron transport between NiO_x_ particles and nanotubes.

In contrast, the SEM micrographs show that the CuO_x_/MWCNTs are characterized by smaller metal-containing particles, uniformly distributed across the nanotubes surface. In accordance with Cu 2p NEXAFS and Cu 1s data, they consist of CuO with small Cu_2_O admixture. As follows from the data of Raman and XPS spectroscopy, a significant distortion of the graphene layer and a large number of sp^3^ hybridized electronic states of carbon atoms associated with adsorbed molecules on nanotubes surface is observed. It can be assumed that the reason for these observations is due to the size of the metal oxide nanoparticles, the nature of their distribution, and chemical bonding on the surface of the nanotube. Namely, all CuO nanoparticles are distributed on the surface of MWCNTs. The theoretical analysis of C 1s XANES spectra confirms that the interaction of CuO_x_ particles with the tube most likely occurs through the formation of Cu–O–C bonds; no Cu–C bond is formed. We assume that, in the case of CuO_x_/MWCNTs, the third type of interaction is realized to a greater extent.

As stated above, complex nanostructures with a non-covalent type of interaction can exhibit greater electrophysical characteristics compared to nanostructures with a covalent type of interaction at the interfaces. In addition, the electrochemical properties of the electrodes improved as the concentration of the carboxylic groups increased. The presence of these two factors can improve the electrochemical properties of the electrodes.

Perhaps this assumption is illustrated by the better electrochemical characteristics of NiOx/MWNTs than CuOx/MWCNTs. Especially considering that the surface area of nanostructures and their porosity hardly play a significant role in this case, since, according to BET analysis (not presented here), the surface area decreases, and the total pore volume increases almost equally for both types of nanostructures.

## 5. Conclusions

In the present investigation, the comprehensive comparative analysis of structure and interfacial interaction in the promising electrode materials NiOx/MWCNTs and CuOx/MWCNTs, obtained by the electrochemical oxidation and dispersion of metal under pulse alternating current, was performed. The investigation was carried out by a number of both surface- and bulk-sensitive experimental and theoretical methods, which made it possible to compile a fairly complete understanding of interfacial interaction in the studied complex nanostructures. The types of interfacial interaction, as one of the possible factors influencing the electrochemical characteristics of nanostructures, have been determined. In NiOx/MWCNTs, a mixed type of interfacial interaction is realized, in which charge transfer occurs through Ni–C and Ni–O–C bonds. The presence of not only Ni–O–C bonds but also non-covalent Ni–C bonds can increase the contact between NiO and MWCNTs, promoting electron transfer between NiO and MWCNTs. The latter is confirmed by the highest value of the calculated adsorption energy (78 meV/atom) in the NiO(200)–CNT interface among the other interfaces, and by the reduction of the tunneling barrier between the nanotube and the top NiO layer by about 2 eV. For NiOx/MWCNTs, better electrochemical characteristics were observed than for the CuOx/MWCNTs where the interfacial interaction is determined by only covalent bonding through Cu–O–C bonds. The specific capacitance of the NiO_x_/MWCNTs is 154, 149, 148 F g^−1^, and 118 F g^−1^ at appropriate current densities of 0.5, 1, 2 A g^−1^, and 5 A g^−1^, in comparison with CuO_x_/MWCNT, where the values of specific capacities are 60.0, 37.0, and 25.5 F/g^−1^, at discharge current densities of 0.5, 1.0, and 1.5 A/g^−1^, respectively. After 1000 charge/discharge cycles, 99% of its initial specific capacity remained, which indicates excellent stability of –NiO_x_/MWCNTs nanomaterial.

## Figures and Tables

**Table 1:** Energy positions and relative contributions of the components in the C 1s XPS spectra of the MWCNT, NiO_x_/MWCNT, and CuO_x_/MWCNT samples.

Sample	Energy Positions, eV					Contribution of the Components, %				
	C=C sp^2^	C–C sp^3^	C–Ox	Ni–C	π–π	C=C sp^2^	C–C sp^3^	C–Ox	Ni–C	π–π
MWCNTs	284.8	285.3	286.3		291.3	88.1	6.6	2.0	–	3.3
NiO_x_/MWCNTs	284.8	285.3	286.3	284.1	291.3	88.4	2.6	3.5	2.3	3.2
CuO_x_/MWCNTs	284.8	285.3	286.3	–	291.3	65.6	8.4	18.2	–	7.7

**Table 2:** Interfaces with maximum adsorption energy.

Interface	Minimal Distance, Å	Adsorption Energy, meV/atom
NiO(200)–CNT	2.66	78
CuO(110)–CNT	3.03	65
Ni(OH)_2_ (001)–CNT	2.37	68
Cu_2_O(110)–CNT	2.98	69

## Data Availability

Data are contained within the article.

## Supplementary Materials

The following supporting information can be downloaded at: https://www.mdpi.com/article/10.3390/nano14110947/s1, Figure S1: Charge density difference isosurfaces for the NiO(200)–CNT interface. Yellow—electron accumulation, Blue—electron depletion; Figure S2: Averaged electrostatic potential along the vertical direction perpendicular to the interface.
