# Supplementary material for: Interfacial Interaction in MeOx/MWNTs (Me–Cu, Ni) Nanostructures as Efficient Electrode Materials for High-Performance Supercapacitors
Source: Nanomaterials (Basel). 2024 May 28;14(11):947. doi: 10.3390/nano14110947 (PMC11173771; doi:10.3390/nano14110947)
Supplement: Supplementary file 1 [file nanomaterials-14-00947-s001.zip › nanomaterials-3010459-supplementary.pdf]

Supplementary information

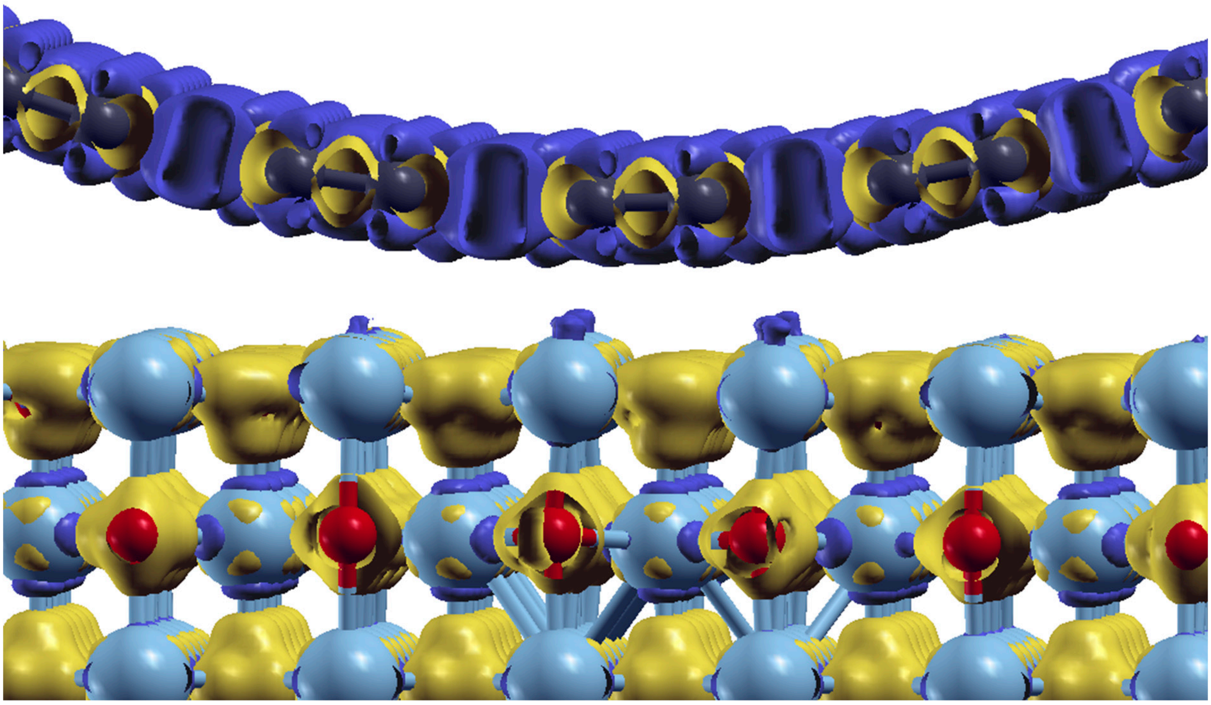

Figure S1. Charge density difference isosurfaces for the NiO(200)-CNT interface. Yellow – electron accumulation, Blue – electron depletion

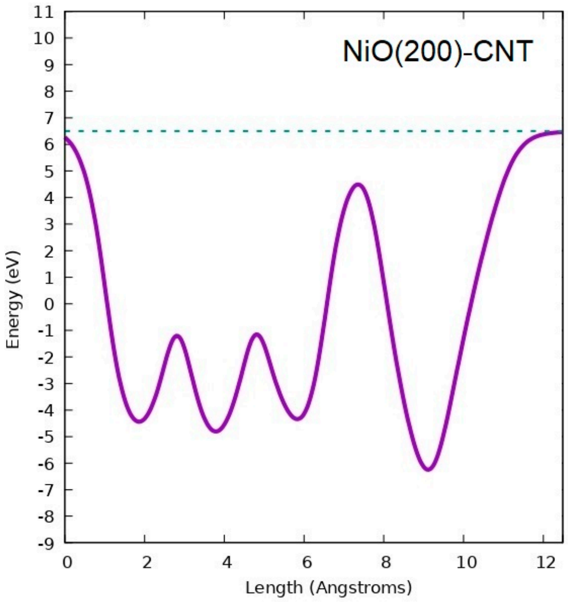

Figure S2. Averaged electrostatic potential along the vertical direction perpendicular to the interface.
